# Genetic parameter estimates for bull prolificacy and its relationship with scrotal circumference in a commercial beef cattle population

**DOI:** 10.1093/tas/txab128

**Published:** 2021-07-29

**Authors:** Chad A Russell, E John Pollak, Matthew L Spangler

**Affiliations:** Department of Animal Science, University of Nebraska-Lincoln, Lincoln, NE 68583, USA

**Keywords:** beef cattle, bull prolificacy, genetic parameters, scrotal circumference

## Abstract

The commercial beef cattle industry relies heavily on the use of natural service sires. When artificial insemination is deemed difficult to implement, multisire breeding pastures are used to increase reproductive rates in large breeding herds or to safe-guard against bull injury during the breeding season. Although each bull might be given an equal opportunity to produce offspring, evidence suggest that there is substantial variation in the number of calves sired by each bull in a breeding pasture. With the use of DNA-based paternity testing, correctly assigning calves to their respective sires in multisire pastures is possible and presents an opportunity to investigate the degree to which this trait complex is under genetic control. Field data from a large commercial ranch was used to estimate genetic parameters for calf count (CC; 574 records from 443 sires) and yearling scrotal circumference (SC; *n* = 1961) using univariate and bivariate animal models. Calf counts averaged 12.2 ± 10.7 and SC averaged 35.4 ± 2.30 cm. Bulls had an average of 1.30 records and there were 23.9 ± 11.1 bulls per contemporary group. The model for CC included fixed effects of age during the breeding season (in years) and contemporary group (concatenation of breeding pasture and year). Random effects included additive genetic and permanent environmental effects, and a residual. The model for SC included fixed effects of age (in days) and contemporary group (concatenation of month and year of measurement). Random effects included an additive genetic effect and a residual. Univariate model heritability estimates for CC and SC were 0.178 ± 0.142 and 0.455 ± 0.072, respectively. Similarly, the bivariate model resulted in heritability estimates for CC and SC of 0.184 ± 0.142 and 0.457 ± 0.072, respectively. Repeatability estimates for CC from univariate and bivariate models were 0.315 ± 0.080 and 0.317 ± 0.080, respectively. The estimate of genetic correlation between CC and SC was 0.268 ± 0.274. Heritability estimates suggest that both CC and SC would respond favorably to selection. Moreover, CC is lowly repeatable and although favorably correlated, SC appears to be weakly associated with CC.

## INTRODUCTION

Natural service multiple-sire breeding pastures are common in the beef industry when artificial insemination is deemed difficult to implement. Multisire breeding pastures enable improved reproduction rates and serve as a means of protecting against bull injury during the breeding season. Although an a priori assumption made by beef cattle producers is that each bull has an equal likelihood of producing calves, there often exists considerable variation in the number of calves sired by bulls despite each having passed a breeding soundness exam (Makarechian and Farid, 1985). Unfortunately, predicting the number of calves that each bull produces is currently not possible. Many factors such as libido and service capacity might affect the number of calves produced per sire. Environmental effects such as social ranking/dominance, age, bull to female ratio, and temperament can affect libido and servicing capacity, but there are also genetic effects present (Chenoweth, 1994; Petherick, 2005). Although there is contradictory evidence on whether bull age significantly affects fertility (Petherick, 2005), the number of calves produced peaks at around 5 yr of age (Van Eenennaam et al., 2014).

Estimates of heritability for sire prolificacy are limited in the literature. A recent example in rams, where prolificacy was defined as log_e_ (number of lambs), reported heritability and repeatability estimates of 0.26 ± 0.12 and 0.40 ± 0.09, respectively (Juengel et al., 2019) illustrating that underlying genetic control of this trait complex exists. Unfortunately, given the fact that this trait expresses later in life, after an animal has become a parent, selection can be impeded due to the delay in phenotypic observations to inform genetic merit estimates (i.e., Estimated Breeding Values). Consequently, an early in life indicator trait would be beneficial to collect if it were relatively easy to garner and reasonably genetically correlated to prolificacy. Bulls with high libido have a higher servicing capacity (Chenoweth et al., 1988), but libido is not evaluated in routine breeding soundness exams. Of the traits tested during physical examinations, scrotal circumference is one of the closest correlated traits to bull fertility (Parkinson, 2004). Consequently, the objectives of this study were to estimate genetic parameters for bull prolificacy and its relationship with yearling scrotal circumference in a multibreed beef cattle population.

## MATERIALS AND METHODS

### Animal Care

Animal care and use protocol were not obtained given all data used herein were from an existing database owned by a commercial entity.

### Animals

Records for this study originated from a commercial ranch with a population of composite animals located in the sand hills of Nebraska. The breed composition of the herd was comprised predominately of Angus, Simmental, Red Angus, and South Devon breeds. This ranch had both seedstock and commercial units whereby the seedstock herds generated replacement animals (bulls and heifers) to be used throughout the ranch. Outside germplasm was restricted to semen from AI sires used in the seedstock herds. In addition to AI matings in the seedstock herds, natural service sires were used in multisire pastures. Breeding season lengths were either approximately 25–30 d (heifers) or 56–70 d (cows). All females were either 2 or 3 yr of age at calving. All bulls passed a breeding soundness exam prior to the breeding season. To enable pedigree formation and to facilitate genetic evaluation, DNA-based parentage testing was employed using a commercially available panel of 96 single nucleotide polymorphisms (SNP). The data for this study included bulls used in multisire breeding pastures in the years 2006–2011, their contemporaries, and their offspring.

**Figure 1.**
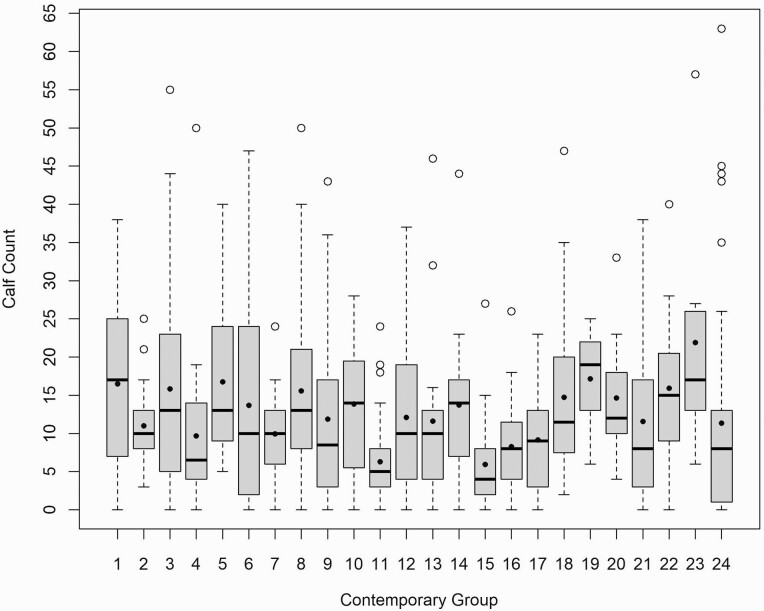
Box and whisker plot of the distribution of calf count in each contemporary group. Contemporary group mean calf count is indicated by black dots.

All AI sires were removed from the data leaving only bulls that had an opportunity to serve as a natural service sire. Additionally, sires were removed if they were not genotyped or were contained in breeding group that produced less than 100 calves. Finally, sires who had counts of calves that were clearly erroneous (greater than 5 SD from the mean over all natural service sires) were also removed. After edits there were 443 unique sires with 574 observations from 24 contemporary groups for further analysis of calf count (CC). Bulls had, on average, 1.30 records for CC and the age at exposure to females was 1.64 ± 0.983 yr. Distribution of CC data by contemporary group is described in Figure 1. Six of the contemporary groups were comprised of exclusively yearling bulls. The remaining groups varied in the age distribution of bulls. Cohort groups were not maintained in subsequent breeding seasons. Yearling scrotal circumference (SC) data were also available on the bulls with CC records and their cohorts without CC records. In total there were 1,961 bulls with SC records representing 9 contemporary groups with an age of 358 ± 54.4 d at measurement. The complete pedigree contained 101,685 animals with 914 sires and 38,898 dams.

### Analysis

All analyses were conducted using the ASREML 4.1 software package (Gilmour et al., 2015). The univariate animal model for CC included fixed effects of age of the animal at exposure (years) and contemporary group (concatenation of breeding pasture and year) and random additive genetic and permanent environment effects, and a residual. The univariate animal model for SC included fixed effects of age (days) and contemporary group (concatenation of month and year of measurement) and random additive genetic effects, and a residual. Breed, and as a consequence, direct heterosis were not fitted in any of the models given breed composition was not known for all animals.

The bivariate animal model included all fixed and random effects from both univariate models to estimate (co)variance components using starting values obtained from the univariate analyses. In matrix notation, the bivariate model can be represented as:


$$\begin{array}{l} \left[\begin{array}{l} y_{CC}\\ y_{SC} \end{array}\right]=\left[\begin{array}{ll} X_{CC} & \;0\\ \;0 & X_{SC} \end{array}\right]\left[\begin{array}{l} b_{CC}\\ b_{SC} \end{array}\right]+\left[\begin{array}{ll} Z_{CC} & \;0\\ \;0 & Z_{SC} \end{array}\right]\left[\begin{array}{l} u_{CC}\\ u_{SC} \end{array}\right]\\ +\left[\begin{array}{ll} W_{CC} & \;0\\ \;0 & \;0 \end{array}\right]\left[\begin{array}{l} p_{CC}\\ \;0 \end{array}\right]+\left[\begin{array}{l} e_{CC}\\ e_{SC} \end{array}\right], \end{array}$$

where *y* is a vector of observations for the traits CC and SC, *X*, *Z* and *W* are incidence matrices relating observations in *y* to levels of fixed effects in *b*, breeding values in *u*, and permanent environmental effects in *p*, respectively, and *e* is a vector of residuals. In the bivariate model, the vector of genetic effects, $u=[u_{1}^{{\;}^{\prime }\;},u_{2}^{{\;}^{\prime }\;}]$, was assumed to be distributed multivariate normal with mean 0 and variance  Φ ⊗A, where ⊗ is the Kronecker product and Φ is the additive genetic (co)variance matrix of CC and SC and A is the numerator relationship matrix. The permanent environmental effects, *p*, were assumed to be distributed as $N\;∼\left(0,\;\sigma_{p}^{2}I_{S}\right)$, where $I_{S}$ was an identity matrix for the number of sires with observations for CC. The vector of residuals, $e=[e_{1}^{{\;}^{\prime }\;},e_{2}^{{\;}^{\prime }\;}]$, was assumed to be distributed multivariate normal with mean 0 and variance $R⊗I_{T}$, where $I_{T}$ was an identity matrix and R was the residual (co)variance matrix.

## RESULTS AND DISCUSSION

### Heritability

Estimates of variance components and their ratios from univariate and bivariate models are reported in Table 1. Univariate heritability estimates for CC and SC were 0.178 ± 0.142 and 0.455 ± 0.072, respectively. Estimates of heritability from the bivariate model for CC and SC were 0.184 ± 0.142 and 0.457 ± 0.072, respectively. Results suggest that although CC is lowly heritable genetic progress could be made for the complex trait of bull prolificacy. However, the estimate from the current study is less than the estimate of 0.26 reported by Juengel et al. (2019) from a sheep population comprised of field data from multiple breeds.

**Table 1.** Estimates of (co)variance components and genetic parameters and their associated standard errors from univariate and bivariate models for calf count (CC) and scrotal circumference (SC)

	Univariate		Bivariate	
	CC	SC, cm	CC	SC, cm
Additive variance	18.7 ± 15.2	1.98 ± 0.342	19.3 ± 15.2	1.99 ± 0.342
Permanent environmental variance	14.4 ± 16.4		14.0 ± 16.3	
Residual variance	71.8 ± 8.62	2.38 ± 0.295	71.6 ± 8.60	2.37 ± 0.294
Heritability	0.178 ± 0.142	0.455 ± 0.072	0.184 ± 0.142	0.457 ± 0.072
Repeatability	0.315 ± 0.080		0.317 ± 0.080	
Genetic correlation			0.268 ± 0.274	
Residual correlation			–0.144 ± 0.132	

The evidence of prolificacy being heritable opens up the possibility of reducing the number of bulls required in breeding pastures to adequately service cows and possibly departing from widely held maxims such as bull to female ratios (BFR) of 1:25 for bulls who have undergone breeding soundness examination (BSE). Bulls that have passed BSE can service more females, meaning that BFR is not the limiting factor and that bulls held to this ratio are inefficient (Chenoweth, 2000). Through increased prolificacy and reduced emphasis on BFR, fewer bulls may be required to attain desired levels of pregnancy. A reduction in the number of bulls required in extensive cattle production enterprises could have a tangible impact on production costs. As shown by Taylor and Field (1995), bull costs per cow are affected by BFR. With a potential reduction in the number of bulls required when BFR is changed, costs related to bull purchase and maintenance could be reduced.

The heritability estimate for SC reported from the current study was higher than the 0.38 estimate reported by Latimer et al. (1982) but similar to Bourdon and Brinks (1986) estimate of 0.49. Differences in the point estimates of heritability among studies can be attributed to differences among the populations used and the models employed. In example, Latimer et al. (1982) fitted breed in the model whereas the current study was not able to and thus the current estimate of heritability for SC could be biased upward. Regardless, the estimate of heritability for SC reported herein was within the range of estimates reported by Koots et al. (1994) who reported a mean of 0.45 across multiple studies.

### Repeatability

Estimates of repeatability for CC from univariate and bivariate models were 0.315 ± 0.080 and 0.317 ± 0.080, respectively. The low repeatability estimate reported in the current study are below the range of 0.43–0.69 reported by Holroyd et al. (2002) using *Bos indicus* animals. The lower estimate of repeatability reported herein could be due to the limited number of repeated records in the population used, as well as, the small number of records for CC (*n* = 574) used in the study. Although literature estimates suggest that a bull’s performance, in terms of number of offspring produced, in one breeding season is predictive of his performance in subsequent breeding seasons the estimate from the current study suggests such a prediction would be lowly accurate. Repeatability is the proportion of phenotypic variation that can be attributed to variation in genetics and permanent environmental effects. Only additive genetic effects were explicitly modeled in the current study. However, the sires were admixed and thus breed and some degree of heterotic effects might be present that were unaccounted for. These unmodeled effects (breed proportion of heterosis) could have influenced the magnitude of the estimate of (narrow sense) heritability and repeatability in the current study. Regardless, the repeatability estimate clearly shows that bull prolificacy comes about through a combination of inherent differences between bulls, unknown permanent environmental effects, and temporary environmental effects (i.e., year).

### Genetic Correlation

The estimate of genetic correlation between CC and SC was low and positive (0.268 ± 0.274) with a large standard error. The point estimate suggests that approximately 7.18% (genetic correlation squared) of the additive genetic variation in CC is shared with SC. Given this estimate, SC could be a valuable indicator of CC at a relatively early age before CC data becomes available. Furthermore, this estimate suggests that indirect improvement in CC could be achieved by selection for increased SC at yearling, albeit inefficient compared to having direct measurements of CC.

### Other Contributing Factors to Prolificacy

Sire prolificacy is a complex trait, and is comprised of several more refined characteristics including male fertility, libido, and the interactions among bulls in the same breeding pasture. When comparing libido measured though testing between rams, high libido sires produced twice the number of progeny than lower libido sires (Stellflug et al., 2006). There does exist limited evidence in beef cattle that libido is heritable. Quirino et al. (2004) reported a heritability estimate of 0.34 for unadjusted libido in a population of Nellore bulls. The same authors reported heritability estimates of 0.31 and 0.19 when libido was adjusted for scrotal circumference and body weight, respectively; interestingly the genetic correlation with SC was moderately negative (−0.43). Libido has also been reported to be favorably genetically correlated to semen volume, motility, and total defects (Quirino et al., 2004). In swine, genetic correlations with number born and sperm motility and sperm abnormalities have produced contradictory results that may be attributed to breed differences (Wolf, 2010). Semen parameters may still be favorably genetically correlated with CC as they are often used during BSE testing and can be used to predict bull fertility (Kastelic and Thundathil, 2008).

Social ranking and dominance are more related to seniority than age or weight (Blockey, 1979), however dominance has been shown to be negatively correlated to libido in yearling bulls (Ologun et al., 1981). This implies that the more dominant bulls may choose to be less prolific, thus negatively impacting overall herd fertility. However, if enough females have entered estrus at the same time, the dominant bull might be unable to keep all the subordinates from mating (Blockey, 1979). Indeed, these social effects themselves could be heritable. Indirect genetic effects (e.g., Bijma, 2014) such as social dominance and aggression could impact interactions among groups of bulls in breeding pastures and thus the prolificacy of certain bulls. In the presence of such indirect genetic effects, selection for improved prolificacy in group mating situations becomes more complex.

## CONCLUSION

The current study suggests that bull prolificacy, as defined by the count of calves sired in multisire breeding pastures, is heritable and would respond to selection. Moreover, estimates suggest this trait complex is lowly repeatable, and thus, performance across successive breeding seasons/years is lowly correlated and thus multiple measurements would enhance accuracy of genetic predictions. Although positively genetically correlated, yearling scrotal circumference does not appear to be a strong indicator of the genetic potential of prolificacy. Given the large standard errors associated with the genetic parameter estimates reported herein, additional studies from larger populations would be beneficial to further quantify the genetic control of bull prolificacy and its relationship with scrotal circumference. As DNA-based paternity assignment becomes more common in commercial settings, this trait complex can be further investigated to bring more resolution to the limited genetic parameter estimates that exist for bull prolificacy.
